# Revising the Subsystem Nurse’s A-Phase-Silicocarnotite within the System Ca_3_(PO_4_)_2_–Ca_2_SiO_4_

**DOI:** 10.3390/ma9050322

**Published:** 2016-04-28

**Authors:** Patricia Ros-Tárraga, Patricia Mazón, Luis Meseguer-Olmo, Piedad N. De Aza

**Affiliations:** 1 Instituto de Bioingenieria, Universidad Miguel Hernandez, Avda. Ferrocarril s/n. Elche, Alicante 03202, Spain; p.ros.tarraga@gmail.com; 2 Departamento de Materiales, Óptica y Tecnologia Electrónica, Universidad Miguel Hernández, Avda. Universidad s/n, Elche (Alicante) 03202, Spain; pmazon@umh.es; 3 Service of Orthopaedic at Arrixaca University Hospital, UCAM-Catholic University of Murcia, Murcia 30120, Spain; lmeseguer.doc@gmail.com

**Keywords:** phase diagram, bioceramic, biocompatibility, calcium phosphate silicate

## Abstract

The subsystem Nurse’s A-phase-silicocarnotite within the system Ca_3_(PO_4_)_2_–Ca_2_SiO_4_ was conducted as a preliminary step toward obtaining new biomaterials with controlled microstructures. Phase composition of the resulting ceramics was studied by X-ray diffraction, differential thermal analysis, and scanning electron microscopy with attached wavelength dispersive spectroscopy. The results showed that the sub-system presents an invariant eutectoid point at 1366 ± 4 °C with a composition of 59.5 wt % Ca_3_(PO_4_)_2_ and 40.5 wt % Ca_2_SiO_4_, and typical eutectoid microstructure of lamellae morphology. These results are in disagreement with the previous reported data, which locate the invariant eutectoid point at 1250 ± 20 °C with a composition of 55 wt % Ca_3_(PO_4_)_2_ and 45 wt % Ca_2_SiO_4_. In addition, cell attachment testing showed that the new eutectoid material supported the mesenchymal stem cell adhesion and spreading, and the cells established close contact with the ceramic after 28 days of culture. These findings indicate that the new ceramic material with eutectoid microstructure of lamellae morphology possesses good bioactivity and biocompatibility and might be a promising bone implant material.

## 1. Introduction

Bioceramics have attracted wide attention due to their cost-effectiveness, easy production, and good biocompatibility. The development of bioceramics has provided promising alternatives to replacing or increasing parts of the skeletal system [1,2]. Therefore, it is significant to develop new type of bioceramics. Compositions belonging to the sub-system Nurse’s A-phase-silicocarnotite within the system Ca_3_(PO_4_)_2_–Ca_2_SiO_4_ are promising candidates for preparing ceramic bone implants.

Nurse’s A-phase is a solid solution with an approximate composition of 7CaO·P_2_O_5_·2SiO_2_, but should not be confused with the mineral of the same composition identified by Nagelshmidt in 1937 [3]. Nurse’s A-phase possess the simple hexagonal crystal lattice of a compound of the type ABXO_4_ where one-eighth of the cations of the hexagonal structure are not occupied [4,5]. Substances with ABXO_4_ structure, particularly solid solutions, with unoccupied cation positions are not unusual. It has been pointed out previously [6,7], that such a deviation from the ideal composition of the structural type is not surprising in view of the high temperatures at which such ABXO_4_ substances are prepared. Nurse’s A-phase, from now on referred to as A, which has been recently synthesized at temperatures around 1550 °C in our laboratory [8,9,10], is a good candidate for biomedical applications [11].

On the other hand, silicocarnotite can be defined as calcium silicophosphate with a carnotite structure. Some authors [12,13] determined that the silicocarnotite structure is very close to hydroxyapatite that has the same weight per unit volume and presents a wide range of CaO, P_2_O_5_, and SiO_2_ solid solutions. Others suggested that the loss of all –OH from HA due to SiO_4_ substitution results in a silicocarnotite structure. They considered that silicocarnotite as a mixture of silicon substituted dehydrated apatite and oxyapatite. Ruys [14] found that silicocarnotite can be present as an impurity in HA structures at the lowest SiO_2_ content accompanied by increasing amounts of α-TCP and β-TCP in the Ca–Si–P–O amorphous phase. Silicocarnotite (5CaO·P_2_O_5_·SiO_2_), from now on referred to as S, is also suitable candidate for biomedical applications. It has been found to be biocompatible and bioactive [15].

The binary system dicalcium silicate (Ca_2_SiO_4_=C_2_S)-tricalcium phosphate (Ca_3_(PO_4_)_2_=TCP) was first studied about 128 years ago when silicocarnotite was described as a component of slag [16]. The system was first studied with X-ray diffraction using a high-temperature stage by Nurse *et al*. [17] They identified two intermediate compounds: 2 Ca_2_SiO_4_–Ca_3_(PO_4_)_2_, referred to as Nurse’s A-phase and silicocarnotite (Ca_2_SiO_4_·Ca_3_(PO_4_)_2_). Below the liquidus there is a continuous high-temperature solid solution at any given ratio of Ca_2_SiO_4_/Ca_3_(PO_4_)_2_ (Rss=α-C_2_S-α-TCP).

The C_2_S-TCP system was also studied by Fix *et al*. [18], who include important modifications to the extension of various solid solution fields. Recently, other authors revised the binary system, including modifications to the invariant eutectoid points [19,20].

The exact knowledge of the phase relationships in the subsystem Nurse’s A-phase-silicocarnotite is essential for designing materials with controlled chemical, mechanical, and biological properties. In the present work, a correction to the phase equilibrium diagram of the system C_2_S-TCP [18] in the sub-system Nurse’s A-phase-silicocarnotite is proposed in the light of experimental data. Phase composition of the resulting ceramics was studied by X-ray diffraction (XRD), differential thermal analysis (DTA), and scanning electron microscopy (SEM) with attached wavelength dispersive spectroscopy (WDS). In addition, the bioactivity and biocompatibility of the new eutectoid ceramics were studied in adult mesenchymal stem cells of human origin (hMSCs-A) cultured in experimental conditions for up to 28 days.

## 2. Results and Discussion

**Figure 1:**
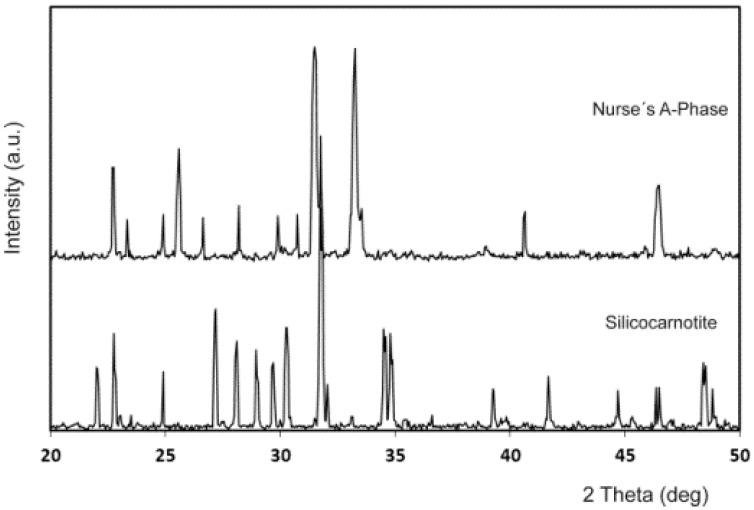
X-ray diffraction patterns of the synthesized materials: Nurse’s A-phase and silicocarnotite.

Figure 1 shows the X-ray diffraction patterns of the synthesized powders of A_ss_ and S_ss_. The obvious sharp peaks and low back grounds suggest that the powders were highly crystalline. The obtained A_ss_ phase shows diffraction peaks that can be assigned to the characteristic reflections of 2Ca_2_SiO_4_·Ca_3_(PO_4_)_2_ (JCPDS card number 011-0676). On the other hand, S_ss_ can be assigned to the characteristic reflections of Ca_2_SiO_4_·Ca_3_(PO_4_)_2_, as were described by Dickens and Brown [21]. The only crystalline phase detected in the sample was a well-crystallized silicocarnotite (JCPDS card number 40-393). As the phases are solid solutions, the diffraction peaks are slightly displaced with respect to the corresponding JCPDS card. The results of the chemical analysis of the materials obtained are displayed in Table 1.

**Figure 2:**
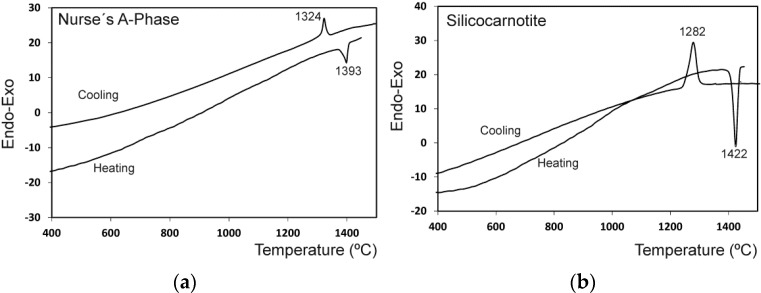
DTA of the Nurse’s A-phase (**a**) and silicocarnotite materials (**b**).

The results of the differential thermal analysis of A_ss_ and S_ss_ are shown in Figure 2. The A_ss_ phase shows only one endothermic peak during heating at 1392 °C that may be assigned to the polymorphic transformation A_ss_ → R_ss_ where R_ss_ phase is a solid solution of α-C_2_S-α′TCP. During cooling, an exothermic peak as a results of the R_ss_ → A_ss_ transformation was observed at a lower temperature that on heating (1324 °C). S_ss_ showed one endothermic peak during heating at 1422 °C that may be assigned to the polymorphic transformation of S_ss_ → R_ss_. During cooling, an exothermic peak as a result of the R_ss_ → S_ss_ transformation was observed at a lower temperature than on heating (~1282 °C).

The experimental results showing the phase coexisting in equilibrium for each composition after the solid state treatment at the selected temperatures and plateau times are summarized in Table 2.

**Figure 3:**
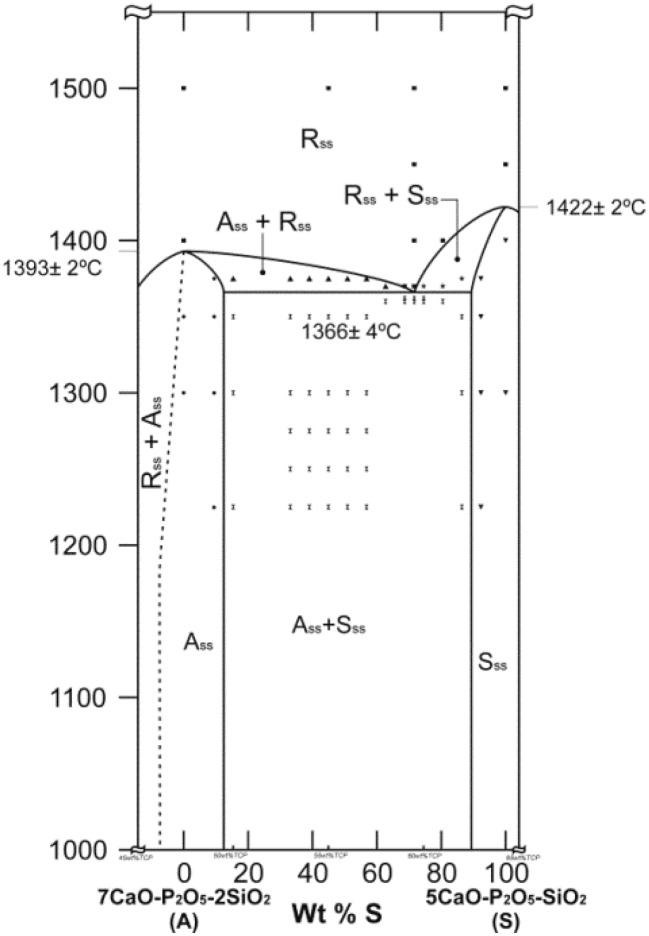
Redrawn of the subsystem Nurse’s A-phase-silicocarnotite within the binary system C_2_S-TCP. (S_ss_: silicocarnotite_ss_; Nurse’s A: Nurse’s A-phase_ss_; R_ss_: (α-C_2_S-α′TCP)_ss_).

The subsystem Nurse’s A-phase-silicocarnotite that was plotted from these results is shown in Figure 3. The markings in the diagram near the eutectoid point indicate the compositions tested in this study.

**Figure 4:**
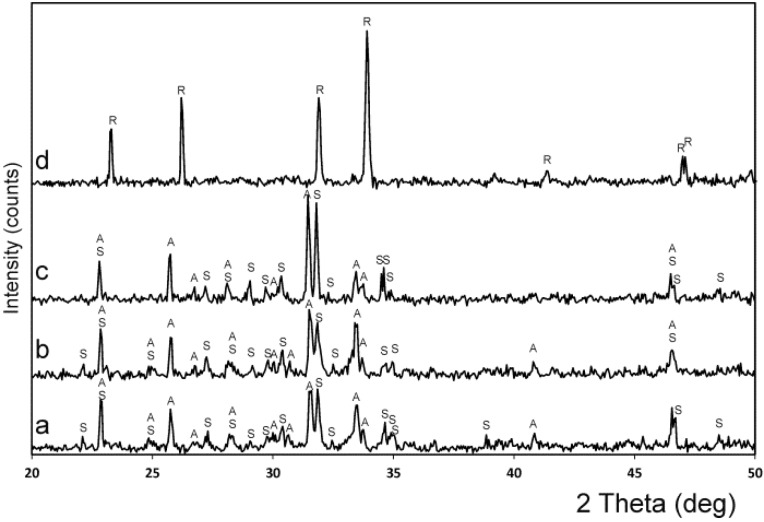
XRD patterns of samples (**a**) 55 wt % A/45 wt % S heated at 1250 °C; (**b**) 55 wt % A/45 wt % S heated at 1275 °C; and (**c**) 28.39 wt % A/71.61 wt % S, heated at 1362 °C; and (**d**) 28.39 wt % A/71.61 wt % S, heated at 1370 °C. (S: silicocarnotite_ss_; A: Nurse’s A-phase_ss_; R: (α-C_2_S-α′TCP)_ss_).

In Figure 4, the X-ray patterns of specimens 55 wt % A/45 wt % S and 28.39 wt % A/71.61 wt % S, obtained at various temperatures are shown. In the sample 55 wt % A/45 wt % S (the invariant point of [18]) Nurse’s A and silicocarnotite phases was identified in this specimen when quenched after the heat treatment at the Fix *et al*. invariant point (1250 °C for 48 h (Figure 4a)) and above the Fix *et al*. invariant point (1275 °C for 48 h (Figure 4b)). On the other hand, below the new invariant point (Figure 4c) the material presents two phases: Nurse’s A and silicocarnotite. Above the new invariant point (Figure 4d) there is only one phase corresponding to the R_ss_ phase, which is a solid solution of α-C_2_S-α′TCP.

**Figure 5:**
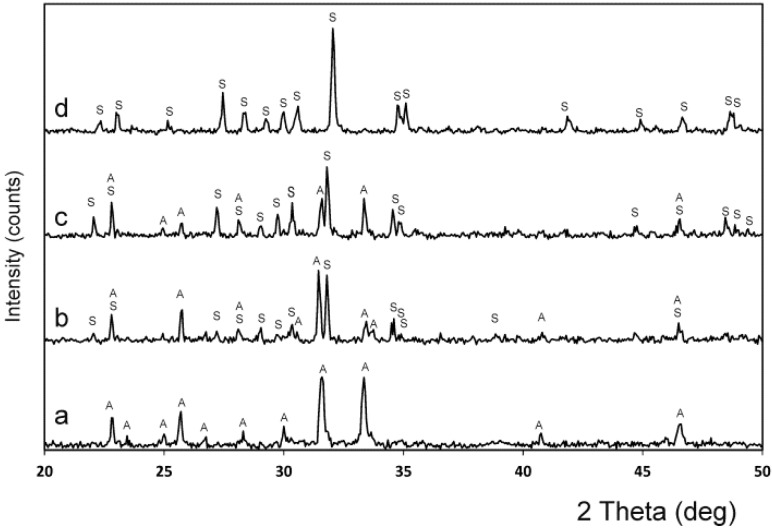
XRD patterns of samples (**a**) 90.48 wt % A/9.52 wt % S; (**b**) 84.57 wt % A/15.43 wt % S; (**c**) 13.60 wt % A/86.40 wt % S; and (**d**) 7.69 wt % A/92.31 wt % S. All of them heated at 1350 °C. (S: silicocarnotite_ss_, A: Nurse’s A-phase_ss_; R: (α-C_2_S-α′TCP)_ss_).

In Figure 5, the X-ray patterns of several compositions obtained after the heat treatment at 1350 °C are shown. Figure 5a,b present the specimens with 90.48 wt % A/9.52 wt % S, and 84.57 wt % A/15.43 wt % S showing the boundary between the phase field of Nurse’s A phase and silicocarnotite + Nurse’s A. Figure 5c,d present the specimens with 13.60 wt % A/86.40 wt % S, and 7.69 wt % A/92.31 wt % S showing the boundary between the phase field of silicocarnotite + Nurse’s A and silicocarnotite_ss_.

In the composition range between 100 wt % A and 100 wt % of S, the high-temperature R phase was obtained at room temperature as a metastable phase. The relevant XRD patterns were essentially of the same type as the XRD spectrum that is shown in Figure 4d. They are characterized by two strong diffraction lines whose separation decreases systematically as the solid solution R become richer in C_2_S. The *d* values for the six strongest lines of the R phase, recorded from the samples quenched from 1500 °C after 144 h, are given in Table 3 together with the d values of the pure α′-TCP (JCPDS card number 89-8960) and α-C_2_S (JCPDS card number 87-1260) for comparison purposes.

**Figure 6:**
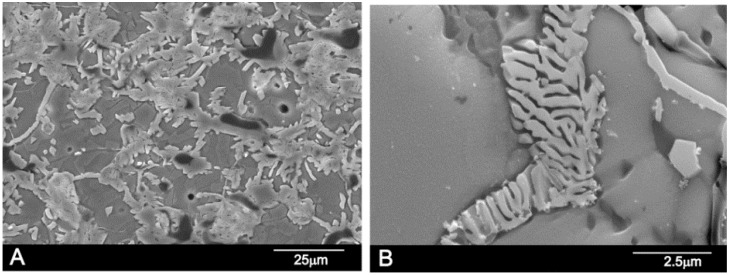
SEM images of sample 55 wt % A/45 wt % S. (**A**) low magnification; (**B**) high magnification.

The microstructure corresponding to the sample 55 wt % A/45 wt % S (Fix *et al*. invariant point) [18] is shown in Figure 6. The sample, after homogenization, was heated at 1250 °C, (the Fix *et al*. eutectoid temperature, 1250 ± 20 °C) for 48 h, then cooled at a rate of 6 °C/min to room temperature. The final microstructure was composed of hypoeutectoid Nurse’s A grains, plus nucleating on grain boundaries, the eutectoid phase composed of Nurse’s A and silicocarnotite due to the decomposition of the R phase.

**Figure 7:**
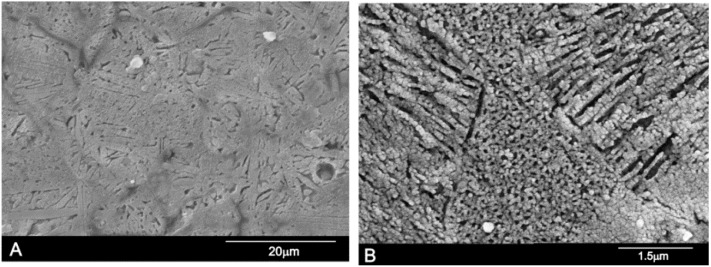
SEM images of sample 28.39 wt % A/71.61 wt % S. (**A**) low magnification; (**B**) high magnification.

The microstructure corresponding to the composition 28.39 wt % A/71.61 wt % S, is shown in Figure 7. The sample, after homogenization, was heated at 1350 °C/48 h and slowly cooled to room temperature at a rate of 6 °C/min. Figure 7a,b shows a typical eutectoid composition of lamellae morphology confirming the location of the eutectoid point. The sample was made up of very thin eutectoid platelets constituted of Nurse’s A and silicocarnotite phases. The two phases were so intimately mixed and thin that it was impossible to distinguish between Nurse’s A and silicocarnotite by WDS.

These results clearly disagree with those previously published by Fix *et al*. [18], who reported that the system shows an invariant eutectoid point at 1250 ± 20 °C with a composition of 55 wt % Nurse’s A and 45 wt % silicocarnotite.

**Figure 8:**
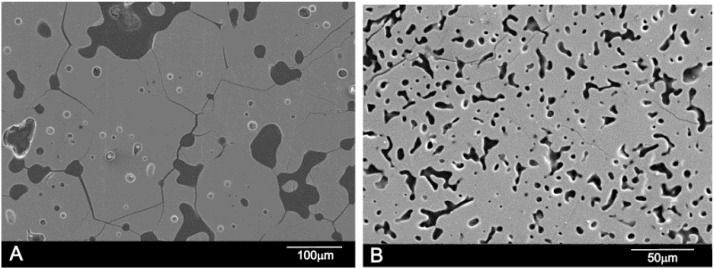
SEM images of sample (**A**) 7.69 wt % A/92.31 wt % S; and (**B**) 90.48 wt % A/9.52 wt % S.

On the other hand, sample 7.69 wt % A/92.31 wt % S at 1350 °C showed a monophasic crystalline microstructure consisting of silicocarnotite_ss_ grains, as shown in Figure 8A. This finding is in good agreement with the XRD results where only the silicocarnotite phase is detected (Figure 5d). Figure 8B shows the polished and etched surface of 90.48 wt % A/9.52 wt % S, at 1350 °C as representative of all compositions in the phase field of Nurse’s A. No significant microstructural features were observed except closed elongated pores of ≈25 µm size. This finding is in good agreement with the XRD results where only Nurse’s A phase was detected (Figure 5a). WDS confirmed the presence of phosphorous, calcium, and silicon.

**Figure 9:**
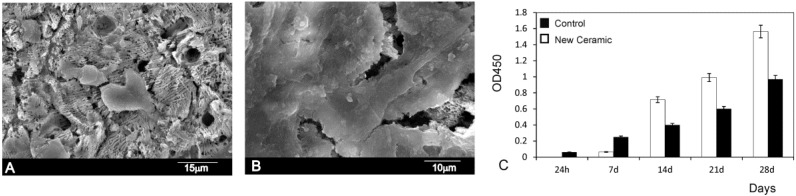
SEM images of the hMSCs-A cultured on eutectoid ceramic surface at (**A**) 24 h; (**B**) 28 days; and (**C**) Proliferation rate of hMSCs-A measured by MTT absorbance at different times of the study.

The results of the *in vitro* cell tests are shown in Figure 9. The morphology of hMSCs-A adhering and spreading on eutectoid ceramic samples after incubation for 24 h and 28 days is shown in Figure 9A,B. In general, the cells appeared flat and adhered well onto the eutectoid ceramic surfaces in all time intervals. The hMSCs-A undergo their morphological changes to stabilize the cell-material interface of the eutectoid ceramic.

The adhesion was enhanced by means of multiple cytoplasmic digitations that spread across the surface of the material, increasing the contact area with the surface material. After 24 h of incubation (Figure 9A) the majority of cells cultured on the material exhibited a round morphology. In this period the cells in the eutectoid ceramics studied presented a similar morphology showing some cells adhering either individually or in small groups dispersed across the surface of the material. After 28 days, the density of cultured cells increased in the bioeutectic material with a marked decrease in the intercellular spaces. At this time point, no signs of cytotoxicity (debris, decreased cell size, sloughed cells, *etc*.) were observed. So that the cells spread and presented a close contact with the material, and the philopodia projected progressively (Figure 9B), which indicates that the ceramic supported hMSCs-A adhesion and spreading.

The hMSCs-A viability assay using the MTT assay (Figure 9C) reveals that the cell proliferation gradually increased being that the percentage of cell viability significantly differed from the negative control group (*p* < 0.05). The hMSCs-A viability assay confirmed the SEM observations.

*In vitro* cell–material interaction is a useful criterion in the evaluation of new biomaterials [22,23,24]. The present study indicates that ceramic supports hMSCs-A cells proliferation. Cells have been found in close contact with the ceramic. This cell behavior suggests that the surface of the eutectoid material is non-irritant and does not affect the structural integrity of the cell. The cells appeared flat and exhibited an intact, well-defined morphology, with cytoplasmic extensions. The preservation of cytoplasmic extensions is important because they allow a vital three-dimensional network within bone.

## 3. Materials and Methods

Samples were prepared from Nurse’s A and Silicocarnotite ceramics which were used as starting materials. Details of the technique and the characterization of the starting materials can be found in previous publications [8,9,10,15].

### 3.1. Chemical Analysis

Chemical analysis of the starting materials was performed in a MagiX Super Q Version 3.0 X-ray fluorescence spectrometer (Philips, Almelo, The Netherlands) provided with an Rh X-ray tube and a power generator of 2.4 kW. Powdered samples weighing 0.3000 g were mixed with 5.5 g of spectral-grade Li_2_B_4_O_7_ and melted in a Pt/Au crucible and formed into disks in a special Perl’X3 (Philips) controlled furnace. Calibration curves were prepared from standards of certified compositions of natural and synthetic calcium silicates.

### 3.2. Phase Diagram Studies

Mixtures with compositions of Table 4 were selected and prepared for studying the subsystem Nurse’s A-phase-silicocarnotite. Batches of each composition weighing ~10 g were prepared. Nurse’s A and silicocarnotite powders were previously dried at 110 °C overnight. The required amounts of each component were weighed in an analytical balance and homogenized three times in an agate mortar with acetone. The resulting pastes were dried at 60 °C, disaggregated in the agate mortar and isostatically pressed at 200 MPa into bars of ~100 mm in length and ~5 mm in diameter. cylinders, ~6 mm in length, were cut from the bars and loaded into small platinum foil crucibles, and then fired at selected temperatures in a Entech-EEFX/17 (Ängelholm, Sweden) equipped with an electronic programmable temperature controller (JUMO Imago 500, Fulda, Germany). The crucibles were suspended in the hot zone of the electrical furnace by a Pt wire and the hot junction of a calibrated Pt/ 6Rh–Pt/30Rh thermocouple was placed very near the crucibles to guarantee the minimum error in the measurement of the temperature of the specimens (±2 °C). Specimens were fired at temperatures ranging from 1225 °C to 1500 °C and time varying from 4 to 144 h (six days) to reach equilibrium. After treatment, the samples were liquid-nitrogen quenched. In addition, duplicate sample were heat treated in the same way, but cooled slowly inside the furnace up to the temperatures within 1275–1225 °C range, which is slightly above and below the eutectoid temperature, as reported by Fix *et al*. (1250 ± 20 °C) [18], and kept at these for 48 h, followed by quenching in liquid nitrogen. The specimens were sometimes reground after quenching and then pressed and fired again to ascertain that the equilibrium had been achieved.

### 3.3. Thermal Analysis

To evaluate the transition temperatures of the Nurse’s A and silicocarnotite, DTA measurements were carried out using a Mettler-Toledo TGA/DTA 851e device (Mettler-Toledo Intl. Inc., Barcelona, Spain). Measurements were performed on ~25 mg of powder, in a platinum crucible, over the range 25–1550 °C, at a heating rate of 5 °C/min, under nitrogen flux. At 1550 °C, the temperature was held constant for 30 min to equilibrate the samples. DTA results established the temperatures at which the crystalline phases formed and their possible polymorphic transformations.

### 3.4. XRD

To evaluate the phase’s composition, XRD patterns were obtained in a Bruker AXS D8-Advance X-ray Diffractometer (Karksruhe, Germany) using λCuKα1 radiation (0.15418 nm) and a secondary curved graphite monochromator. Diffractograms of samples were compared with the data provided by the Joint Committee on Powder Diffraction Standards (JCPDS) database. JCPDS cards numbered as 011-0676, 40-393, 89-8960, and 87-1260, were used for Nurse’s A, silicocarnotite, α′-tricalcium phosphate, and α-dicalcium silicate, respectively.

### 3.5. Microstructure and Microanalysis

Equilibrated samples were embedded in an epoxy resin under vacuum and progressively polished down to 0.1 µm with diamond paste and etched with acetic acid with a concentration of 0.5% for 2 s. Then, they were gently cleaned in an ultrasonic bath with distilled water, dried, and palladium-coated for scanning electron microscope (SEM-Hitachi S-3500N, Ibaraki, Japan) observations. The chemical composition of crystalline grains was qualitatively determined with a wavelength dispersive spectroscopy (WDS) system coupled to the above-described electron microscope.

### 3.6. In Vitro Cell Tests

Adult mesenchymal stem cells (hMSCs-A) were isolated from the bone marrow of adult human volunteers obtained through direct aspirations of the iliac crest. The hMSCs-A were seeded in 75 cm^2^ flasks at a density of 5 × 10^3^/cm^2^ and were cultured in α-Minimum Essential Medium (α-MEM) supplemented with 10% fetal bovine serum (FBS) and antibiotics penicillin/streptomycin (100 µg/mL and 100 µg/mL, respectively). The cells were cultured at 37 °C in a 5% CO_2_ atmosphere and 95% relative humidity, making a change of medium every 3–4 days. When the culture was confluent, the subculture was treated with 0.25% w/v Trypsin-EDTA in sterile phosphate-buffered saline (PBS; pH 7.4) for 5 min. For this study only the hMSCs-A at the third Nurse’s A passage were employed. The increment in the number of cells on the materials was evaluated through the reduction of a tetrazolium salts (MTT) assay using the method described previously [25,26] and plastic as a control.

The surface morphology of samples was analyzed by SEM-WDS in order to evaluate the cell growth and adhesion to the ceramics surface. After incubation for 12 h and 7, 15, 21, and 28 days, the samples were removed from the culture well, rinsed with PBS and fixed with 3% glutaraldehyde in 0.1 M cacodylate buffer for 1.5 h at 4 °C. Then, they were rinsed and post-fixed in osmium tetroxide for 1 h, before being dehydrated through increasing concentrations of ethanol (30, 50, 70, and 90 vol %) with final dehydratation in absolute alcohol. After this, they were dried by the critical-point method, palladium-coated, and examined by SEM-WDS.

### 3.7. Statistical Analysis

Data were analyzed by one-way analysis of variance (ANOVA) followed by Tukey-Kramer multiple comparisons. In both analyses, the minimum acceptable level of significance was *p* < 0.05.

## 4. Conclusions

The subsystem Nurse’s A-phase-silicocarnotite within the C_2_S-TCP system presents an invariant eutectoid point at 1366 ± 4 °C for a composition of of 59.50 wt % Ca_3_(PO_4_)_2_ and 40.50 wt % Ca_2_SiO_4_,. At the temperature of the invariant point the maximum content of solid solution of TCP in Nurse’s A is less than ~2.5 wt % and that of C_2_S in silicocarnotite is ~5.5 wt %.

In light of the new findings, the composition and processing of ceramic materials belonging to the subsystem Nurse’s A-phase-silicocarnotite may be designed with a more exact control of final phase composition, microstructure, and properties. The *in vitro* cell test supports the hypothesis that the eutectoid ceramic obtained displays *in vitro* bioactivity and biocompatibility, which makes it a potential candidate for surgical applications.

## Figures and Tables

**Table 1:** Results of the X-ray fluorescence chemical analysis of Nurse’s A and silicocarnotite synthetic powders.

Chemical Analysis (wt %)	A_ss_	S_ss_
CaO	59.8	57.9
SiO_2_	18.3	11.9
P_2_O_5_	21.5	30
MgO	0.064	0.062
Al_2_O_3_	0.084	0.066
Na_2_O	0.038	0.028
K_2_O	0.007	0.005
Fe_2_O_3_	0.036	0.032
TiO_2_	0.024	0.007
MnO	–	–

**Table 2:** Phases identified by XRD for each composition after the heat treatment at the selected temperatures and plateau times. (S_ss_: silicocarnotite_ss_; A_ss_: Nurse’s A-phase_ss_; R_ss_: (α-C_2_S-α′TCP)_ss_; * Fix *et al*. invariant point.)

Composition No.	Holding Conditions		Observed Phase	General Characteristics
	Temperature (°C)	Time (h)		
1	1300	144	A_ss_	White solid
	1350	144	A_ss_	White solid
	1400	144	R_ss_	White solid
	1500	144	R_ss_	White solid
2	1225	144	A_ss_	White solid
	1300	144	A_ss_	White solid
	1350	144	A_ss_	White solid
	1375	144	A_ss_	White solid
3	1225	144	A_ss_ + S_ss_	White solid
	1300	144	A_ss_ + S_ss_	White solid
	1350	144	A_ss_ + S_ss_	White solid
	1375	144	A_ss_ + R_ss_	White solid
4	1225	48	A_ss_ + S_ss_	White solid
	1250	48	A_ss_ + S_ss_	White solid
	1275	48	A_ss_ + S_ss_	White solid
	1300	48	A_ss_ + S_ss_	White solid
	1350	144	A_ss_ + S_ss_	White solid
	1375	144	A_ss_ + R_ss_	White solid
5	1225	48	A_ss_ + S_ss_	White solid
	1250	48	A_ss_ + S_ss_	White solid
	1275	48	A_ss_ + S_ss_	White solid
	1300	48	A_ss_ + S_ss_	White solid
	1350	144	A_ss_ + S_ss_	White solid
	1375	144	A_ss_ + R_ss_	White solid
6 *	1225	48	A_ss_ + S_ss_	White solid
	1250	48	A_ss_ + S_ss_	White solid
	1275	48	A_ss_ + S_ss_	White solid
	1300	48	A_ss_ + S_ss_	White solid
	1350	144	A_ss_ + S_ss_	White solid
	1375	144	A_ss_ + R_ss_	White solid
	1500	144	R_ss_	White solid
7	1225	48	A_ss_ + S_ss_	White solid
	1250	48	A_ss_ + S_ss_	White solid
	1275	48	A_ss_ + S_ss_	White solid
	1300	48	A_ss_ + S_ss_	White solid
	1350	144	A_ss_ + S_ss_	White solid
	1375	144	A_ss_ + R_ss_	White solid
8	1225	48	A_ss_ + S_ss_	White solid
	1250	48	A_ss_ + S_ss_	White solid
	1275	48	A_ss_ + S_ss_	White solid
	1300	48	A_ss_ + S_ss_	White solid
	1350	144	A_ss_ + S_ss_	White solid
	1375	144	A_ss_ + R_ss_	White solid
9	1362	144	A_ss_ + S_ss_	White solid
	1370	144	A_ss_ + R_ss_	White solid
10	1362	144	A_ss_ + S_ss_	White solid
	1370	144	R_ss_	White solid
11	1362	144	A_ss_ + S_ss_	White solid
	1370	144	R_ss_	White solid
	1400	144	R_ss_	White solid
	1450	144	R_ss_	White solid
	1500	144	R_ss_	White solid
12	1362	144	R_ss_ + S_ss_	White solid
	1370	144	A_ss_ + S_ss_	White solid
13	1362	144	A_ss_ + S_ss_	White solid
	1370	144	A_ss_ + S_ss_	White solid
	1400	144	S_ss_	White solid
14	1225	48	A_ss_ + S_ss_	White solid
	1300	48	A_ss_ + S_ss_	White solid
	1350	144	A_ss_ + S_ss_	White solid
	1375	144	R_ss_ + S_ss_	White solid
15	1225	48	S_ss_	White solid
	1300	48	S_ss_	White solid
	1350	144	S_ss_	White solid
	1375	144	S_ss_	White solid
16	1300	144	S_ss_	White solid
	1400	144	S_ss_	White solid
	1450	144	R_ss_	White solid
	1500	144	R_ss_	White solid

**Table 3:** *d* values for the six strongest lines of the phase R quenched at 1500 °C after 144 h, together with the *d* values of the pure α′-TCP (JCPDS card number 89-8960) and α-C_2_S (JCPDS card number 87-1260) for comparison purposes. Compositions are in wt %.

C_2_S	TCP	*T* (°C)	Composition No.											
			–		1		6		11		16		–	
			*d*	I	*d*	I	*d*	I	*d*	I	*d*	I	*d*	I
100	0	1525	–	–	3.99	–	3.99	30	4.00	30	3.98	40	3.98	41
52.61	47.39	1500	3.67	4	–	–	3.77	10	3.80	10	3.81	17	3.85	50
45	55	1500	2.92	61	3.75	74	2.95	80	2.94	100	2.94	100	2.96	100
40.50	59.50	1500	2.77	100	2.75	100	2.72	70	2.72	70	2.70	60	2.68	68
35.70	64.30	1500	2.28	9	2.19	22	2.20	16	2.21	8	2.22	22	2.22	17
0	100	1447	2.01	28	2.00	–	2.00	40	1.99	10	1.95	22	1.99	43

**Table 4:** Composition of the samples.

Composition No.	Sample wt % TCP	Sample wt % C_2_S	Sample wt % S	Sample wt % A
1	47.39	52.61	0.00	100.00
2	49.00	51.00	9.52	90.48
3	50.00	50.00	15.43	84.57
4	53.00	47.00	33.18	66.82
5	54.00	46.00	39.09	60.91
6	55.00	45.00	45.00	55.00
7	56.00	44.00	50.92	49.08
8	57.00	43.00	56.83	43.17
9	58.00	42.00	62.74	37.26
10	59.00	41.00	68.66	31.34
11	59.50	40.50	71.61	28.39
12	60.00	40.00	74.57	25.43
13	61.00	39.00	80.48	19.52
14	62.00	38.00	86.40	13.60
15	63.00	37.00	92.31	7.69
16	64.30	35.70	100.00	0.00
